# Typhus Epidemics

**Published:** 1920-02-14

**Authors:** 


### TYPHUS EPIDEMICS.

### Their Potentialities and Limitations.

The present epidemic of typhus which is raging in the Balkan States, in Vienna, and, as far as can be gathered from the strictly partisan news sent out by the Moscow wireless, in Russia, has not unnaturally caused many persons to fear that with the return of the pre-war facilities of travel and intercommunication in Europe a pandemic may arise embracing the whole of the civilised world. With the example of the recent influenza wave still in their minds many imagine a somewhat similar state of affairs in which typhus takes the place of influenza, and the former disease has, on account of its unfamiliarity, come to be looked upon as one of the most consistently fatal, most insidious, and least amenable to treatment of all the scourges which may afflict mankind.

This being so, it may be useful to review our present knowledge on typhus, and also, for the sake of example, to compare it with influenza, and with plague.

### A Comparison.

To begin with, what is the likelihood of a pandemic comparable to the late influenza? Two etiological facts are sufficient to put our fears to rest. Whatever may be the causal organism of typhus, the carrier is the body louse, that much is certain, and the body louse is not an adventurous spirit. His home is man, and he is by nature unable to stray far from his home, nor does he show much desire to do so. In crowded dug-outs, in crowded jails, and in the crowded hovels of the very poor, where the only warmth is got by packing humanity tightly, he may and will move from host to host, and so infest immense numbers of people, and under these conditions typhus may spread with the louse. But there is no air-borne infection, as must be the case in influenza, nor are there the two carriers, the agile flea, and the wandering rat, as is the case with bubonic plague. So, though we may receive numbers of visitors from infected countries, there is not any great fear of infection, unless contact between them and ourselves is very close.

Secondly, though this really arises out of the previous statement, typhus, as some of its older names, as "jail fever" and "famine fever" imply, is a disease of squalor. Sufficiently close contact to allow of the migration of the louse is the prime factor in infection, and general debility, resulting from privation, starvation, and want, is the controlling factor as regards to susceptibility.

It was stated by Hilton Fagge, at a time when typhus was fairly prevalent in England, that out of 18,000 cases over a period of twenty-three years 95.7 per cent. had been inmates of hospitals, or dependent on parish relief. And the case of Serbia, where there has been no peace since 1911, and where the land has been desolated by three successive invasions, is in no way comparable to that of the more fortunate countries of Western Europe.

However, even though cases should occur in this country, they could be dealt with with much greater ease and certainty of success than influenza or plague. A thorough disinfestation, the methods of which are only too well known to those who have served in the Army, will free the patient and the "contacts," their clothes and belongings, and lastly their house, from any lice. And, again, there is no rat to act as the Wandering Jew was said to act with regard to cholera—to leave a trail behind him. Nor are the results of disinfestation as unreliable as were those obtained by disinfecting the air passages for influenza, which in some hands worked wonders, in others not so.

### The Mortality.

Then again, the mortality in typhus is, in normal communities, somewhere about 20 per cent., and only rises to a greater percentage when combined with famine. On the other hand, the mortality of influenza is variously given and is much less dependent on existing debility in the subject, while plague has a death-rate of from 30 to 95 per cent., which again is almost independent of the general state of health of its subjects. Another factor which will decrease the likelihood of typhus being introduced is that it is a disease of the poor, and it is unlikely that any but the rich will be able to indulge in extensive travel for a year or so, except in so far as emigrants may move, and they are subjected to such examinations and restrictions that any cases could easily be detected, and the contacts disinfected before any great degree of harm had occurred.

### Diagnostic Means.

The diagnosis of typhus, too, is now no longer a case for the clinician alone. The Weil-Felix test, an agglutination reaction similar to the Widal reaction for typhoid, in which a special strain of *Bacillus Proteus* replaces *Bacillus typhosus abdominis*, is now accepted on all hands as being pathognomonic of the disease, and this though the organism in question is not in any way the causal organism of typhus. It may be remarked in parentheses that rumour has it that when this reaction was discovered by the Germans in about 1917, the whole arts of our secret service were trained on to obtaining a culture of the particular strain of *B. Proteus* required, and that ultimately it was obtained in Bulgaria. With this test in our hands there should be no difficulty in detecting mild cases once any suspicions were aroused, and once detected the rest is easy.

On the whole, then, we may take it that the chance of typhus coming to this country is small, that should it come it can be dealt with comparatively easily, that the mortality is less than in influenza, and it is less easily spread—in fact, that a typhus epidemic on a large scale can only come if famine leads the way.

